# Supplementary material for: Insertion-sequence-mediated mutations both promote and constrain evolvability during a long-term experiment with bacteria
Source: Nat Commun. 2021 Feb 12;12:980. doi: 10.1038/s41467-021-21210-7 (PMC7881107; doi:10.1038/s41467-021-21210-7)
Supplement: Supplementary file 1 — Supplementary Information [file 41467_2021_21210_MOESM1_ESM.pdf]

SUPPLEMENTARY INFORMATION

Insertion Sequence-mediated mutations both promote and constrain evolvability during a long-term experiment with bacteria

Jessika Consuegra, Joël Gaffé, Richard E. Lenski, Thomas Hindré, Jeffrey E. Barrick, Olivier Tenaillon, and Dominique Schneider

Supplementary Table 1 Effect of IS elements on population fitness.

		Generations as factor				Generations				Power law generations			
		All	Mutator status	Non-mutators	No IS effect	All	Mutator status	Non-mutators	No IS effect	All	Mutator status	Non-mutators	No IS effect
Number of IS	Effect	-0.0082	-0.0074	-0.008	NA	-0.0076	-0.0058	-0.0072	NA	-0.0074	-0.0066	-0.0064	NA
	p-value	$< 2x10^{-16}$	$< 2x10^{-16}$	$< 2x10^{-16}$	NA	$<2x10^{-16}$	$8.42x10^{-9}$	$3.87x10^{-11}$	NA	$<2x10^{-16}$	$8.42x10^{-9}$	$1.77x10^{-13}$	NA
Mutator status	Effect	NA	0.022	NA	0.087	NA	0.056	NA	0.105	NA	0.027	NA	0.086
	p-value	NA	0.11	NA	$3.1x10^{-11}$	NA	0.000393	NA	$2.99x10^{-13}$	NA	0.0509	NA	$3.12x10^{-11}$

Multivariate regression explaining population fitness taking into account generations (as factors, or numbers transformed or not with a power law), mutator status (all populations, all populations with information on their mutator status, or non-mutator populations), and the number of IS elements. The effect of the accumulation of IS elements on fitness and the associated *p*-values are presented for each model. The impact of being mutator on fitness is also presented in combination with the IS number or without that information.

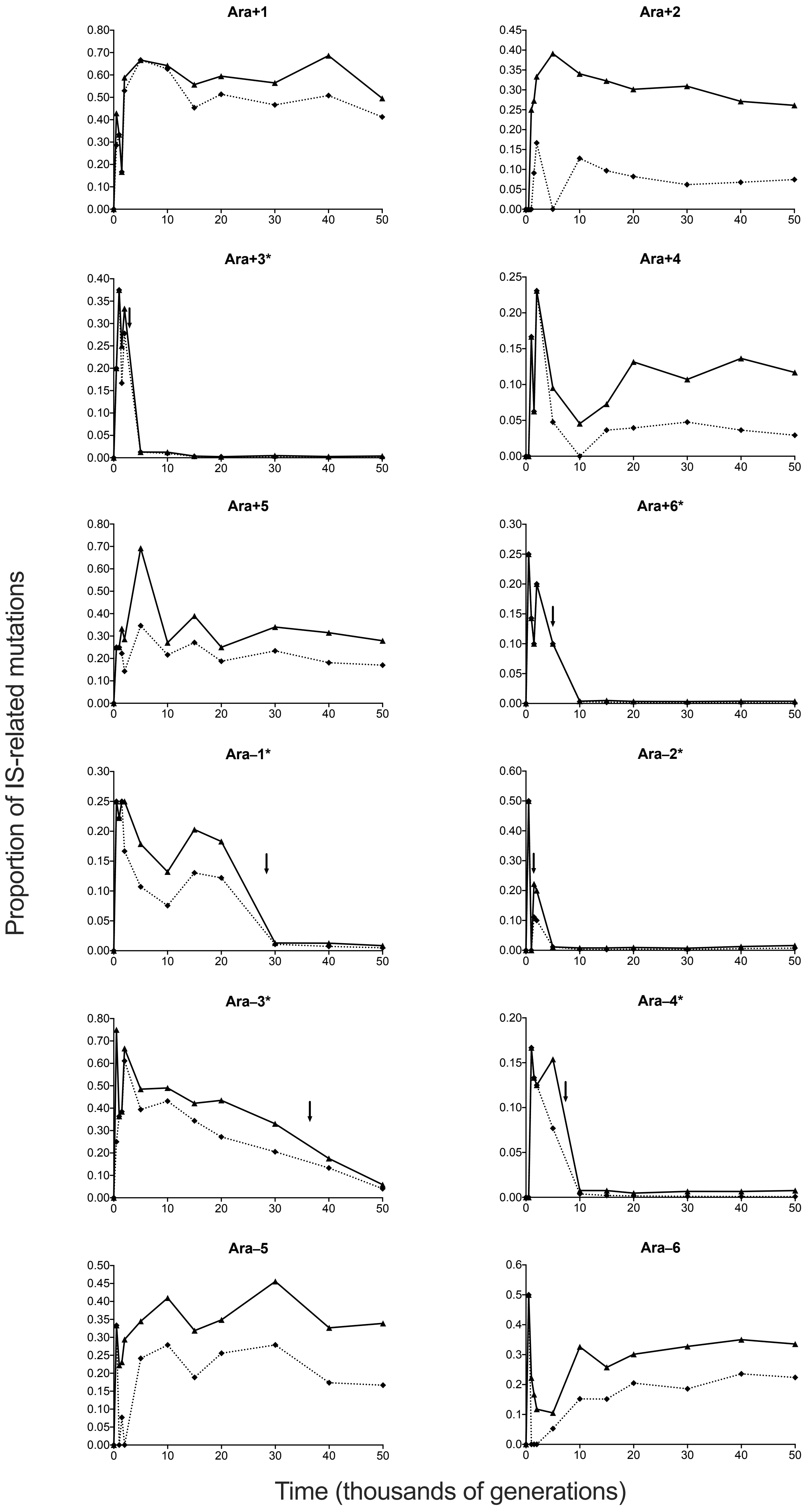
Ara+1
Ara+2
Ara+3*
Ara+4
Ara+5
Ara+6*
Ara–1*
Ara–2*
Ara–3*
Ara–4*
Ara–5
Ara–6
Proportion of IS-related mutations
Time (thousands of generations)

Supplementary Figure 1 Dynamics of the proportion of IS-related mutations relative to total mutations in each of the 12 populations of the LTEE over 50,000 generations. The asterisks indicate the six populations that evolved point-mutation hypermutability. The arrows indicate the approximate time points at which these populations became hypermutators. Triangles, plain line: all IS-related mutations. Diamonds, dotted line: IS*150*-related mutations. Source data are provided as a Source Data file.

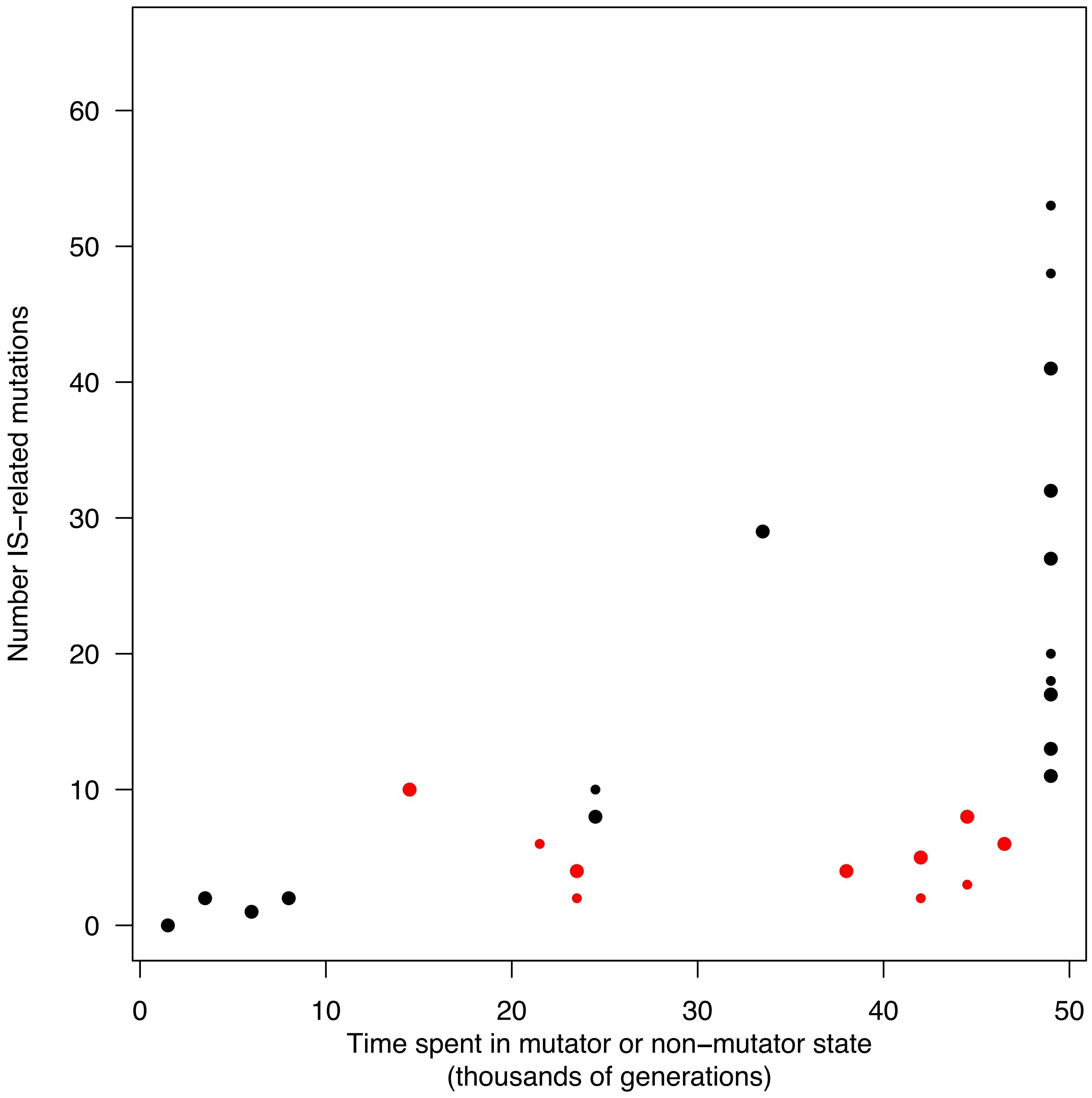
Number IS–related mutations
0
10
20
30
40
50
60
0
10
20
30
40
50
Time spent in mutator or non–mutator state
(thousands of generations)

Supplementary Figure 2 Number of fixed IS-related mutations as a function of the time in mutator or non-mutator state. Large and small dots show mutations fixed in major and minor lineages, respectively. (Both include mutations fixed in the total population, so they are not fully independent). This analysis is the same as shown in Fig. 4, except here we exclude mutations that appeared within 1000 generations before or after the mutator status changed. Red and black dots indicate mutator and non-mutator populations, respectively. Source data are provided as a Source Data file.

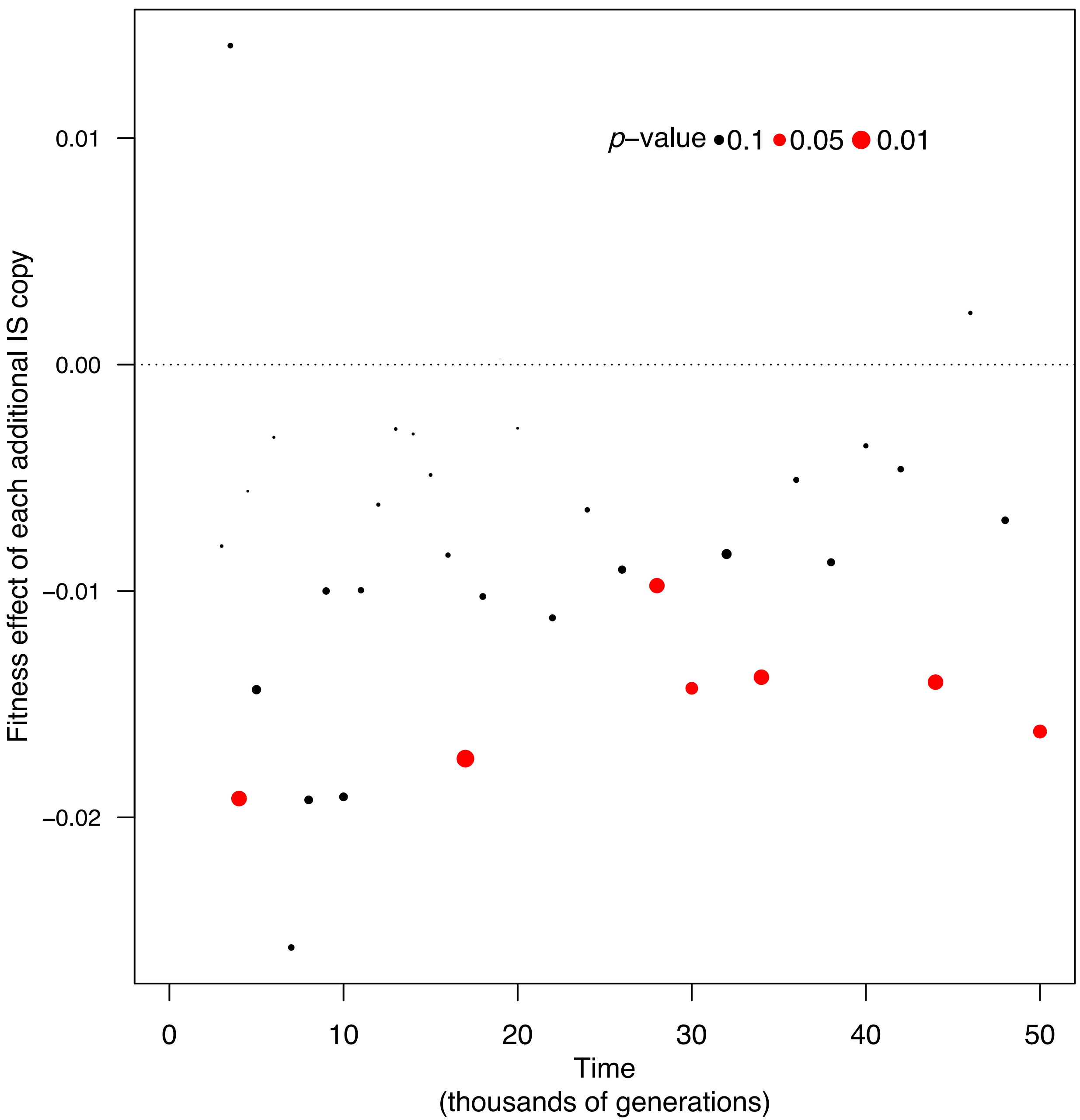

p–value
0.1
0.05
0.01
0.01
0.00
–0.01
–0.02
Fitness effect of each additional IS copy
0
10
20
30
40
50
Time
(thousands of generations)

Supplementary Figure 3 Relationship between fitness and IS-related mutations. Multiple regression analysis to estimate the marginal fitness effect (the cost, when negative) associated with having an extra IS copy. This analysis is the same as shown in Fig. 5, except here we do not take into account mutator status. The size of the points is larger for lower *p*-values; red points indicate a significant contribution of IS copy number to fitness at the 5% level. Source data are provided as a Source Data file.

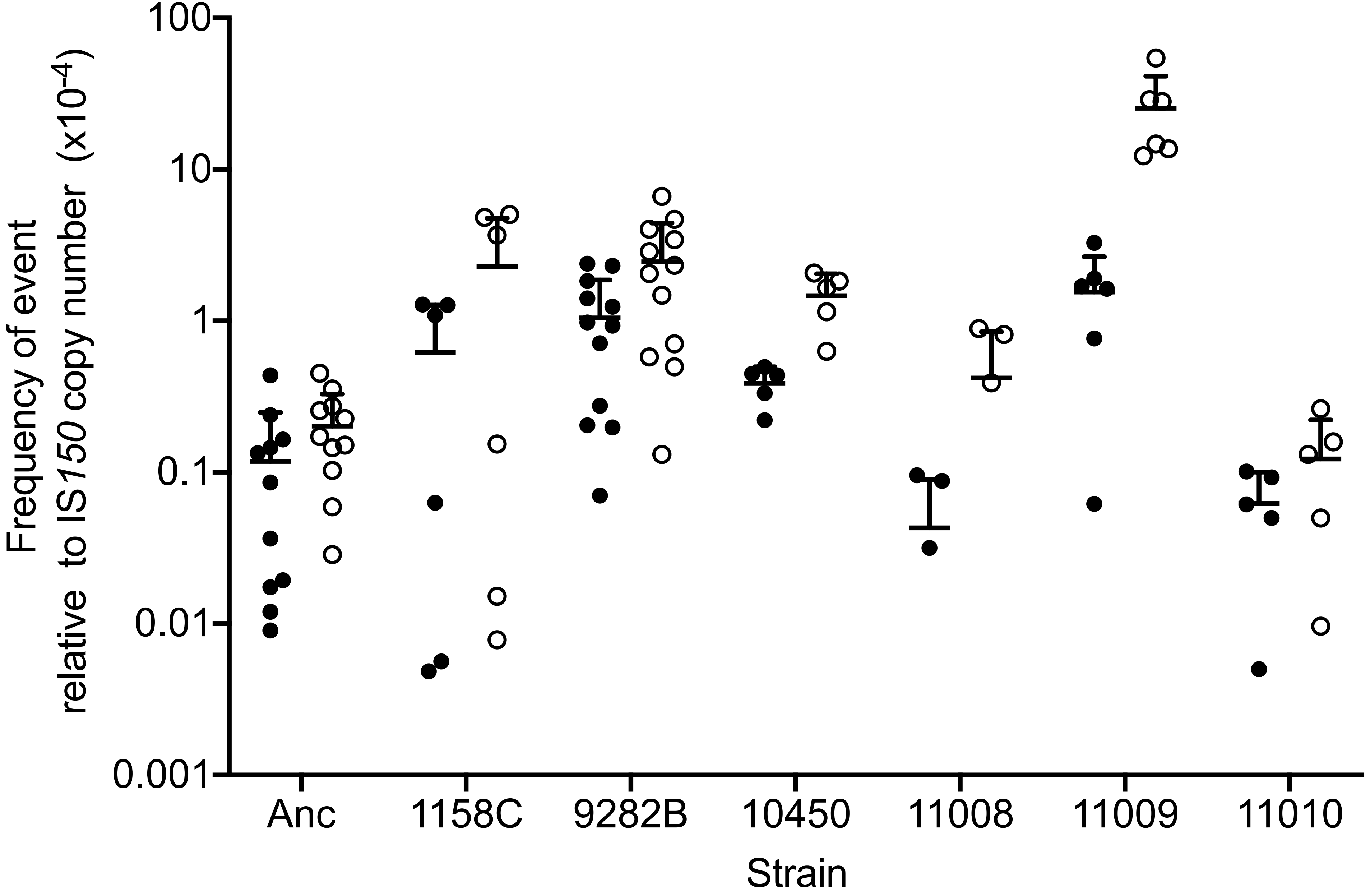

Frequency of event
relative to IS150 copy number (x10-4)
100
10
1
0.1
0.01
0.001
Anc
1158C
9282B
10450
11008
11009
11010
Strain

Supplementary Figure 4 IS*150*-mediated recombination and transposition events in the ancestor and evolved clones from population Ara+1. Ratio of IS*150*-mediated transposition (black) and recombination (white) events, expressed relative to chromosomal copy number (obtained by dividing the respective frequencies in Fig. 6b by the copy number in Fig. 6a). Error bars show the standard deviation based on three technical replicates for each of three biological replicates, except for the ancestor which had three technical replicates for each of five biological replicates and 1158C and 11009 which had three technical replicates for each of two biological replicates. The generation at which each evolved clone was sampled is shown in parentheses below the strain identifier for each evolved clone; Anc denotes the ancestral strain. Source data are provided as a Source Data file.
