# Supplementary material for: Insertion-sequence-mediated mutations both promote and constrain evolvability during a long-term experiment with bacteria
Source: Nat Commun. 2021 Feb 12;12:980. doi: 10.1038/s41467-021-21210-7 (PMC7881107; doi:10.1038/s41467-021-21210-7)
Supplement: Supplementary file 3 — Description of Additional Supplementary Files [file 41467_2021_21210_MOESM3_ESM.pdf]

Description of Additional Supplementary Files

File Name: Supplementary Data 1
Description: Number of copies of the 12 different IS element families in the ancestor and two evolved clones sampled at each of 11 generations from the 12 LTEE populations (separate Excel file).
