# Supplementary material for: Insertion-sequence-mediated mutations both promote and constrain evolvability during a long-term experiment with bacteria
Source: Nat Commun. 2021 Feb 12;12:980. doi: 10.1038/s41467-021-21210-7 (PMC7881107; doi:10.1038/s41467-021-21210-7)
Supplement: Supplementary file 5 — Reporting Summary [file 41467_2021_21210_MOESM5_ESM.pdf]

nature research

Corresponding author(s): Schneider Dominique

Last updated by author(s): Dec 24, 2020

Reporting Summary

Nature Research wishes to improve the reproducibility of the work that we publish. This form provides structure for consistency and transparency in reporting. For further information on Nature Research policies, see our Editorial Policies and the Editorial Policy Checklist.

Statistics

For all statistical analyses, confirm that the following items are present in the figure legend, table legend, main text, or Methods section.

n/a	Confirmed	
☐	☒	The exact sample size (*n*) for each experimental group/condition, given as a discrete number and unit of measurement
☐	☒	A statement on whether measurements were taken from distinct samples or whether the same sample was measured repeatedly
☐	☒	The statistical test(s) used AND whether they are one- or two-sided<br>*Only common tests should be described solely by name; describe more complex techniques in the Methods section.*
☒	☐	A description of all covariates tested
☐	☒	A description of any assumptions or corrections, such as tests of normality and adjustment for multiple comparisons
☐	☒	A full description of the statistical parameters including central tendency (e.g. means) or other basic estimates (e.g. regression coefficient) AND variation (e.g. standard deviation) or associated estimates of uncertainty (e.g. confidence intervals)
☐	☒	For null hypothesis testing, the test statistic (e.g. *F*, *t*, *r*) with confidence intervals, effect sizes, degrees of freedom and *P* value noted<br>*Give P values as exact values whenever suitable.*
☒	☐	For Bayesian analysis, information on the choice of priors and Markov chain Monte Carlo settings
☒	☐	For hierarchical and complex designs, identification of the appropriate level for tests and full reporting of outcomes
☐	☒	Estimates of effect sizes (e.g. Cohen's *d*, Pearson's *r*), indicating how they were calculated

Our web collection on statistics for biologists contains articles on many of the points above.

Software and code

Policy information about availability of computer code

Data collection	No software was used for data collection.
Data analysis	Microsoft Excel (versions 2013 and 2016) was used for data analyses. The softwares Breseq (18. Tenaillon, O. et al. Tempo and mode of genome evolution in a 50,000-generation experiment. Nature 536, 2016, 165–170) and Civi (version 1.0) were used for genomic analyses. Regression models using the function lm from R software (RStudio version 1.1.453 and R version 3.6.2 2019-12-12) were used for analyzing the relationship between IS elements, mutator status and fitness.<br>Analysis scripts for this study have been deposited to the Dryad Digital Repository (doi:10.5061/dryad.m37pvmd0v).

For manuscripts utilizing custom algorithms or software that are central to the research but not yet described in published literature, software must be made available to editors and reviewers. We strongly encourage code deposition in a community repository (e.g. GitHub). See the Nature Research guidelines for submitting code & software for further information.

Data

Policy information about availability of data

All manuscripts must include a data availability statement. This statement should provide the following information, where applicable:

- Accession codes, unique identifiers, or web links for publicly available datasets
- A list of figures that have associated raw data
- A description of any restrictions on data availability

The datasets produced by this study have been deposited (41. Consuegra, J et al. IS-mediated mutations both promote and constrain evolvability during a long-term experiment with bacteria. Dryad, https://doi.org/10.5061/dryad.m37pvmd0v (2020)) to the Dryad Digital Repository (doi:10.5061/dryad.m37pvmd0v). Source data are provided with this paper.

Field-specific reporting

Please select the one below that is the best fit for your research. If you are not sure, read the appropriate sections before making your selection.

- [x] Life sciences
- [ ] Behavioural & social sciences
- [ ] Ecological, evolutionary & environmental sciences

For a reference copy of the document with all sections, see nature.com/documents/nr-reporting-summary-flat.pdf

Life sciences study design

All studies must disclose on these points even when the disclosure is negative.

Sample size	No sample size calculation was performed. The number of biological replicates was choosen (>=3) to ensure reproducibility.
Data exclusions	No data exclusions
Replication	For the measurement of IS150 transposition frequencies: The experiment was performed in three independent biological replicates containing each three technical replicates. The results were consistent among biological replicates
Randomization	Ramdomization is not relevant to our study since it involves molecular genetics, microbiology and genome sequences analyses
Blinding	No relevant to our study. Indeed, a single researcher executed each experiment

Reporting for specific materials, systems and methods

We require information from authors about some types of materials, experimental systems and methods used in many studies. Here, indicate whether each material, system or method listed is relevant to your study. If you are not sure if a list item applies to your research, read the appropriate section before selecting a response.

Materials & experimental systems

n/a	Involved in the study	
x		Antibodies
x		Eukaryotic cell lines
x		Palaeontology and archaeology
	x	Animals and other organisms
x		Human research participants
x		Clinical data
x		Dual use research of concern

Methods

n/a	Involved in the study	
x		ChIP-seq
x		Flow cytometry
x		MRI-based neuroimaging

Animals and other organisms

Policy information about studies involving animals; ARRIVE guidelines recommended for reporting animal research

Laboratory animals	No laboratory animals
Wild animals	No wild animals
Field-collected samples	No field-collected samples
Ethics oversight	No ethics oversight needed

Note that full information on the approval of the study protocol must also be provided in the manuscript.
